# Field evaluation of electrophysiologically-active dung volatiles as chemical lures for trapping of dung beetles

**DOI:** 10.1038/s41598-023-50079-3

**Published:** 2024-01-05

**Authors:** Nisansala N. Perera, Russell A. Barrow, Paul A. Weston, Leslie A. Weston, Geoff M. Gurr

**Affiliations:** 1 Gulbali Institute for Agriculture, Water and Environment, Charles Sturt University, Wagga Wagga, NSW 2678 Australia; 2 School of Agriculture, Environment and Veterinary Sciences, Charles Sturt University, Wagga Wagga, NSW 2678 Australia; 3 School of Agriculture, Environment and Veterinary Sciences, Charles Sturt University, Orange, NSW 2800 Australia

**Keywords:** Zoology, Ecology

## Abstract

Dung beetles are economically important beneficial insects that process dung. To locate this source, they use volatile organic compounds (VOCs). The objectives of the study were to evaluate the attractiveness of ten electrophysiologically-active dung volatiles (phenol, skatole, indole, *p*-cresol, butanone, butyric acid, eucalyptol, dimethyl sulphide, dimethyl disulphide, and toluene) to dung beetles in the field and to investigate how the composition of volatile blends influences efficacy as lures for use in traps. Six combinations of the compounds were compared with field collected cattle dung bait and a negative control, across three seasons. Both dung and synthetic baits captured all exotic dung beetle species present in the study area. A six-compound mix (M1), comprising major dung volatiles, served as an attractive chemical mixture. The addition of dimethyl sulphide, dimethyl disulphide (M2) and toluene (M4) enhanced attractancy of M1 for dung beetles, while eucalyptol (M3) decreased the attractancy. The degree of attraction by various dung beetle species to synthetic baits varied, but baits proved to be effective, especially for summer trapping. The trap design used in this study presented a convenient and practical way to sample dung beetle and other associated scarabs from open pastures. The attraction of introduced dung beetle species to synthetic baits is documented here for the first time in Australia. In addition, necrophagous *Omorgus* sp. is reported here for the first time to be attracted to synthetic baits. They showed a significant attraction to the mixture containing dimethyl sulphide and dimethyl disulphide (M2). The current study represents a promising first step towards formulating a synthetic chemical lure for dung beetles, offering a consistent, standardised, and bio-secure trapping method compared to use of naturally occurring dung baits, especially as a multi-species lure.

## Introduction

Dung beetle communities are a characteristic of a healthy terrestrial ecosystem. In addition to dung burial that cycles nutrients in grazing ecosystems, dung beetles are biological control agents suppressing pests and parasites^1^ and bioindicators of anthropogenic activities that impact on biodiversity^2^. The structure of dung beetle communities at a given location depends on factors such as vegetation cover, climatic conditions, and soil conditions^3^. Dung beetles are attracted to fresh dung, a process mediated by dung volatile organic compounds (VOCs) detected with their antennae^4–7^. Numerous reports have documented the efficacy of fresh dung baits to trap dung beetles^4,8,9^, but the types of VOCs emitted by fresh dung vary with the host diet, the sex of the animal, the gut microbiome and the secretions of prior colonising insects^10–14^. Preliminary experiments suggest that freezing of dung for later use may also alter the headspace volatile composition which in turn may impact dung attractiveness. Given the spatial and temporal variation of dung beetle attraction to a particular dung bait, use of dung baits as lures is not likely to be a reliable measure of species richness and abundance of the existing dung beetle fauna in a given area. Furthermore, dung baits may also present biosecurity risks due to the potential for the unintentional spread of invasive species and parasites, as well as the transmission of diseases.

Various chemicals have recently been tested in the field to gain an improved understanding of the ecological basis of the attraction of dung beetles to dung VOCs^4,9^. Major dung VOCs tested in the field in earlier studies include indole, skatole, phenol, butyric acid, butanone and *p*-cresol. Skatole^4^ and butyric acid^9^ were more attractive among European dung beetle assemblages as individual compounds. Through electroantennography, it was found that these compounds can trigger nerve impulses in the antennae of certain dung beetle species^10,15,16^. Screwworm (*Cochliomyia hominivorax*) lure, which included butyric acid, phenol, *p*-cresol, dimethyl disulphide and indole in a mixture with several other compounds^17^ efficiently captured a larger number of dung beetles than did traps baited with volatile fatty acids that mimic cow entrails or citronella^18^. Further, some species of dung beetle show a dietary shift towards other food sources such as carrion, mushrooms, rotting fruits or eggs, from which volatiles that are common to dung are released^19–25^. Similarly, benzaldehyde, 2-methyl-1,4-benzoquinone and 2-methoxy-3-methyl-1,4-benzoquinone emitted by defensive glands of diplopods, can attract scarab beetles^21,26,27^. Moreover, speculations exist as to the potential involvement of butanone, cresol, indole, skatole and butyric acid in the burial of decomposing hen eggs by dung beetles^24^.

Adult beetles use a blend of compounds rather than a single compound to locate ephemeral dung pats^4,9,10^. Previous studies indicate that certain dung beetle species exploit a specific blend of volatiles to choose among available dung types^6,19,28^. Even though hundreds of VOCs are present in the dung headspace, only a subset of compounds are likely to be behaviourally active. In general, some of the headspace compounds may act as resource-indicating compounds, while others could be background cues either with a masking effect, enhancing effect or null effect^29,30^. Amidst numerous irrelevant compounds in the surrounding environment, olfactory receptors in beetle antennae need to be able to detect messenger compounds even at low concentrations^31–33^. Due to a lack of experimental studies, the role of dung VOCs in the attraction of dung beetles remains poorly understood.

In Australia, the dung beetle fauna consists of native and exotic species. The native dung beetle population in Australia consist of ca. 500 species^34^. Before European colonisation, the largest terrestrial herbivores in Australia were marsupials, which produce dry, hard dung pellets to which native dung beetles are adapted. When European colonists arrived in 1788, exotic livestock species such as cattle, sheep and horses were brought to Australia, resulting in an ecological imbalance. Because of the adaptation of native dung beetles to marsupial dung, large, wet dung pats produced by introduced livestock started accumulating in pastures, which resulted in fouling of pastures and explosive growth of flies and other pests that breed in dung^7,35,36^. Thus, dung beetle importation programmes commenced in 1964 in order to improve pasture quality and reduce the abundance of nuisance flies^36^. According to the review by Pokhrel et al.^37^, deliberate dung beetle introductions in Australia have occurred over six decades and so far, the number of established exotic species currently stands at over 30^38,39^.

The exotic dung beetle community in the Riverina region of New South Wales as reported by Dung Beetle Ecosystem Engineers project sightings, consists of 11 principal species: *Bubas bison* (a winter active species), *Digitonthophagus gazella*, *Onthophagus taurus*, *O. binodis*, *Onitis alexis*, *O. aygulus*, *Euoniticellus africanus*, *E. fulvus*, *E. intermedius*, *E. pallipes* (summer active species) and *Aphodius fimetarius* (late spring-early autumn active species)^40^. This assemblage presents a valuable research opportunity to investigate the attractiveness of VOCs to introduced dung beetles. In southern Australia, lack of dung beetle activity for 2–3 months, which frequently occurs in late winter/early spring, and can lead to a significant reduction of dung burial and pasture health by 17–25 % in cattle pastures^41^. Therefore, efforts have been made to identify geographical and seasonal gaps in distribution of dung beetles via long-term monitoring programs. Once gaps have been identified, the spread of exotic dung beetles can be accelerated by collecting beetles from areas where they are abundant and relocating them to regions of lower abundance. Currently, dung is used as the bait in traps to survey and collect dung beetles for redistribution. However, dung baits vary in their quality, consistency, moisture content and the VOCs they emit. If synthetic baits were developed and validated, they would offer greater consistency, making them preferable in research studies because of their known and consistent VOC profiles. The aim of the current study was to evaluate the attractiveness of synthetic blends of dung VOCs that include previously screened electroantennographically-active (EAG-active) compounds as potential replacements for dung baits. Field trapping was conducted over three seasons; winter, spring and summer to assess the attractancy of synthetic baits to a range of exotic dung beetle species with varying profiles of seasonal activity. Inclusion of previously unexplored compounds such as eucalyptol, toluene, dimethyl sulphide (DMS) and dimethyl disulphide (DMDS) marked a novel advancement for dung beetle attraction in a field study. Also, this was the first time that chemical compounds were tested in the field for dung beetle attraction in Australia. From an ecological perspective, this experimentation will improve our knowledge on the potential significant role of these volatiles in olfactory resource location by introduced dung beetles. From an applied standpoint, the development of standardised trapping lures or baits that replace dung serves a useful purpose as synthetic baits are better suited for monitoring, given their consistency in contrast to use of dung baits. We aim to understand the dynamics of dung beetle distribution and abundance across southern Australia, to design a standardised surveillance tool for dung beetles.

## Materials and methods

### Field experiment setup

**Figure 1:**
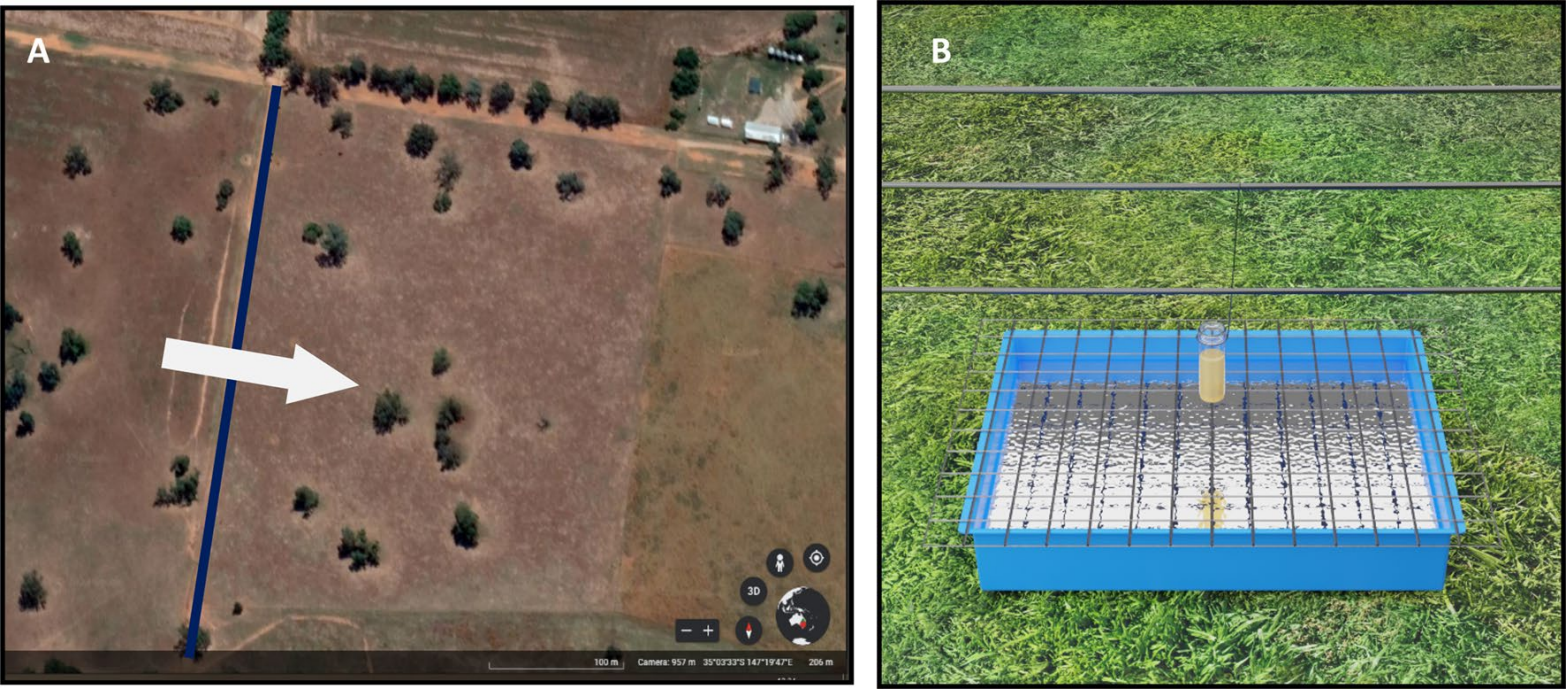
(**A**) Aerial image of the field site illustrating the placement of the transect (blue line) (Charles Sturt University, Wagga Wagga, NSW, Australia). The direction of the prevailing wind is indicated by an arrow. (**B**) Image of a synthetic baited trap (for dung baited traps, vials are replaced with 50 g of fresh dung). Traps consisted of a tray half filled with water. Wire mesh covering the tray and water to prevent beetles from flying away and inhibit predation.

All experiments were performed at the Charles Sturt University Research Farm, Wagga Wagga, NSW (35°03′33″ S–147°19′47″ E) (Fig. 1A), from June 2022 to June 2023 during four trapping sessions using various synthetic lures. The adjacent paddocks had active cattle and occasional sheep populations during the time of the experiment. We selected open pasture environments to not to disrupt air currents or impede beetle flight^42^. Traps were arranged in the laneway between two paddocks and the transect was perpendicular to the prevailing wind to allow odour plumes to be produced emanating from each unique source (Fig. 1A). Traps were placed 10 m apart^4^ and bait treatments were randomly distributed along the transect. Baits of the none of the adjacent traps were of the same type.

### Trap design and baits

The trap design was comprised of a tray (36 × 23 × 7 cm), half filled with water. A wire mesh secured over the tray acted as a barrier to ingress by other animals and provided a surface to secure the bait (Fig. 1B). This trap design was successful in trapping different species of dung beetles during our monitoring work. No preservatives were mixed with the water. These water traps provide low-cost and time-saving beetle sampling method especially for dung beetles. Trap baits were of three types: dung bait as the positive control, attractant-free water trap as the negative control and six combinations of synthetic baits. For dung baits (50 g), dung from pasture-fed cattle was collected fresh before apparent colonisation by insects and kept at 4 °C during the duration of the experiment. Care was taken to collect dung from animals that had not been treated with antiparasitic drugs at least 6 weeks before sampling. The candidate chemicals were selected based on our previous electroantennography study which identified these ten compounds as EAG-active for *B. bison, G. spiniger* and *O. aygulus*^43^ and purchased from Sigma-Aldrich, Australia (Table S2). For synthetic baits, 0.5 mL of liquid components and 0.5 g of solid compounds were used^4,9^. Six different combinations (Table 1) of 10 EAG-active dung VOCs were used as synthetic baits in this experiment.

**Table 1:** Combinations of dung volatiles used as synthetic baits in the field study.

Mix	Content
M1	Skatole + indole + phenol + butyric acid + butanone + *p*-cresol
M2	M1 + dimethyl sulphide + dimethyl disulphide
M3	M1 + eucalyptol
M4	M1 + toluene
M5	M1 + dimethyl sulphide + dimethyl disulphide + eucalyptol + toluene
M6	Dimethyl sulphide + dimethyl disulphide + eucalyptol + toluene

A vial containing the synthetic bait was attached to the fence line, 10 cm above the centre of the relevant trap and dung bait was placed on top of a tissue paper on the mesh. The number of beetles captured in each trap was recorded, and each bait was replaced at 24 h intervals. Four sessions of trapping were performed: winter (20th–24th, June 2022 and 12th–16th, June 2023), spring (29th November–3rd December 2022), and summer (01st–05th February 2023). Day-active beetles (spring through autumn) were targeted by exposing the traps during the daytime from 8:00 h for 24 h, whereas night active beetles (winter) were sampled by setting the traps from 15:00 h to 10.00 h the following morning. Beetle identification was performed using the dung beetle guide^44^ available and using the information on DBEE website^40^. Trapped beetles were counted, identified and then released at a location > 1 km away from the trapping transect.

### Qualitative headspace volatiles analysis of the cattle dung baits

Dung used in the study was analysed using headspace solid-phase micro extraction (HS-SPME). SPME fibres (Agilent Technologies Ltd., USA) were preconditioned in the autosampler heater before volatile adsorption using the manufacturer’s recommended time and temperature, 270 °C for one hour. A sample of 0.7 g of the cattle dung, was placed into a 10 mL screw-capped headspace vial with a sealed aluminium cap (PTFE-lined silicone septum) (Agilent Technologies Ltd., USA). A polydimethylsiloxane/divinylbenzene (PDMS/DVB) fibre with 65 µm phase thickness fitted to an AOC-5000 autosampler system (Shimadzu, Duisburg, Germany) was exposed to the fresh dung headspace for the extraction of volatile compounds. Sampling was carried out for 30 min. Six dung sample replicates for each season were analysed.

Loaded SPME fibres were analysed by gas chromatography/ quadrupole time of flight-mass spectrometry (GC/QToF-MS) equipped with HP-5MS column (30 m × 250 µm inner diameter × 0.5 µm film thickness, Agilent Technologies Ltd., USA). Helium was used as the carrier gas at a flow rate of 1.5 mL/min. Each fibre was immediately desorbed into the ultra-inert straight SPME liner (0.75 mm, Agilent Technologies Ltd., USA) and thermal desorption was performed for 1 min at 250 °C in splitless mode. The initial column temperature was 40 °C for 2 min and then ramped from 40 to 230 °C at a rate of 4 °C/min and subsequently from 230 to 260 °C at a rate of 10 °C/min. Mass spectra were recorded after exposing the GC effluent to electron ionisation (EI) at 70 eV. Mass spectra were collected at a rate of 5 spectra/s with an acquisition time of 200 ms/spectrum over a *m/z* range of 50–500 amu. Total ion chromatograms (TICs) were analysed using chromatogram deconvolution algorithm in Agilent MassHunter (V. 7). The identities of the major VOCs were confirmed by comparing them with standards injections (Sigma-Aldrich, Milwaukee, WI, USA) and through matches of their mass spectra against those in the NIST database (version 2.3, 2017).

### Data analysis

Data was tested for normality using the Shapiro-Wilk test. As data were not normally distributed, non-parametric Wilcoxon two-sample paired test was used to compare bait attractiveness between dung baits and synthetic baits^4^ for each session. Rarefaction analysis for species richness and abundance based on hill numbers (q = 1) was done using iNEXT, an online R based version^45,46^. As raw data did not fulfil the requirements for parametric statistics, number of beetles caught among treatments were analysed by Kruskal-Wallis One-Way Nonparametric ANOVA followed by Dunn’s All-Pairwise Comparisons test which allowed us to determine significant differences between bait types based on their ranks^47,48^. The attractiveness of synthetic lures was compared in relation to the volatile composition of the corresponding dung baits used.

## Results

### The attractiveness of dung and synthetic baits

**Figure 2:**
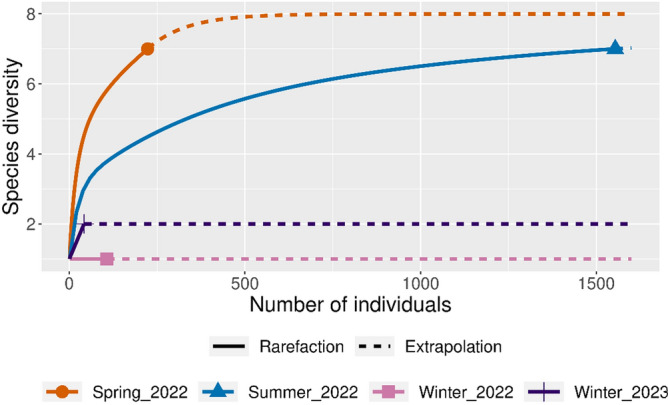
Rarefaction curve showing the cumulative number of dung beetle species collected (total of all treatments) with their total abundance in all four sessions. Line was produced at 95% confidence Interval.

A total of 1926 dung beetles were collected during four trapping sessions (over 18 total trapping days). They belonged to 10 species from the subfamily Scarabaeinae, with eight of them being exotic species and two being native species (Tables 2, S3). The exotic species included *Bubas bison, Onthophagus taurus, Euoniticellus pallipes, E. fulvus, E. africanus, Aphodius fimetarius, Onitis alexis,* and *Digitonthophagus gazella,* and the two native species were *O. pentacanthus* and *O. dandalu*. Among the 10 species trapped, two functional groups were identified; nine species were paracoprids (tunnelers), while the other species was an endocoprids (dweller). Most of the species were represented by a small number of individuals of each, the exception being *O. taurus*, the dominant species trapped. The winter season returned a single species in traps*, B. bison*, which comprised 7.74 % of the total captures. On the other hand, both spring and summer trapping were dominated by *O. taurus,* comprising 82.55% of the total capture (Tables 2, S3). Rarefaction curves indicated that species richness was highest during spring, while the abundance of dung beetles collected was greatest during summer (Fig. 2). Of the 1926 individuals captured, only four beetles were caught in unbaited traps. The total number of dung beetles attracted to baits fluctuated throughout sampling periods, with the greatest number of beetles collected during the summer (December–February) (Fig. S1, Table S3). However, rarefaction curves approached an asymptote indicating that sampling can be considered adequate for further statistical inference (Fig. 2).

**Table 2:** Dung beetle species and *Omorgus* sp. captured (total of all treatment). Exotic dung beetle species are depicted as ‘^E^’ and native species are depicted as ‘^N^’.

Species	Functional group	Winter (2022)	Winter (2023)	Spring	Summer	%
*Bubas bison*^*E*^	Paracoprid	107	41	1	0	7.74
*Onthophagus taurus*^*E*^	Paracoprid	0	0	181	1409	82.55
*Euoniticellus pallipes*^*E*^	Paracoprid	0	0	10	5	0.78
*Euoniticellus fulvus*^*E*^	Paracoprid	0	0	3	59	3.22
*Euoniticellus africanus*^*E*^	Paracoprid	0	0	0	1	0.05
*Aphodius fimetarius*^*E*^	Endocoprid	0	0	8	0	0.42
*Onitis alexis*^*E*^	Paracoprid	0	0	0	2	0.10
*Digitonthophagus gazella*^*E*^	Paracoprid	0	0	19	5	1.25
*Onthophagus pentacanthus*^*N*^	Paracoprid	0	1	1	0	0.10
*Onthophagus dandalu*^*N*^	Paracoprid	0	0	0	73	3.79
*Omorgus* sp. (Family Trogidae)	–	0	0	109	10

**Figure 3:**
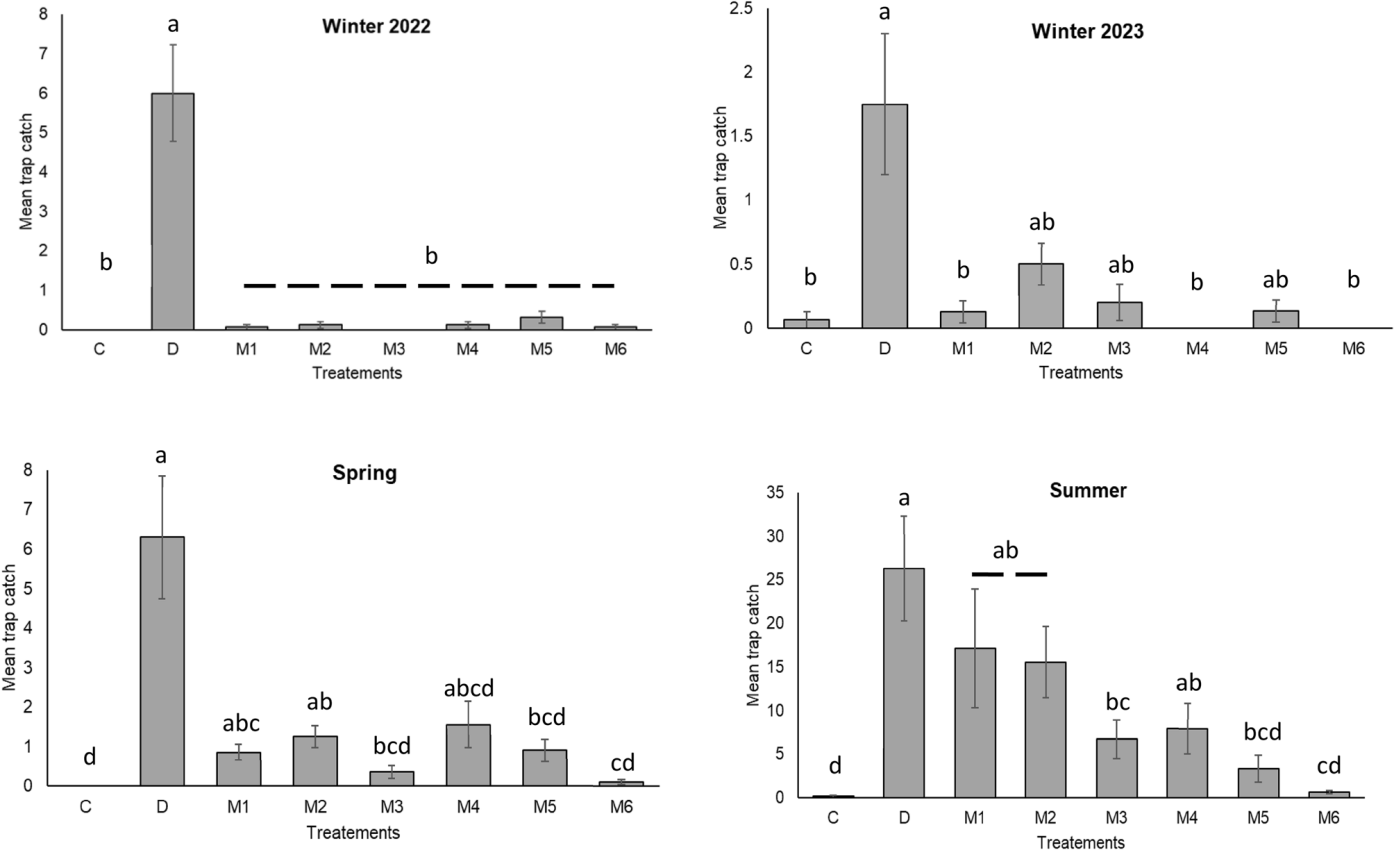
Mean trap catch (± SE) in winter, spring, and summer (Kruskal–Wallis, Dunn ‘s test). For all four sessions *P* < 0.001 as determined by Kruskal–Wallis one-way nonparametric analysis of variance. Bars accompanied by the same letters are not significantly different as determined by Dunn's all pairwise comparison test*.* Refer to Table S3 for species composition.* n* = 4*.* C—bait free, D—dung bait, M1—skatole + indole + phenol + butyric acid + butanone + *p*-cresol*,* M2—M1 + dimethyl sulphide + dimethyl disulphide*,* M3—M1 + eucalyptol*,* M4—M1 + toluene*,* M5—M1 + dimethyl sulphide + dimethyl disulphide + eucalyptol + toluene*,* M6—dimethyl sulphide + dimethyl disulphide + eucalyptol + toluene.

Mean trap catch for both dung and synthetic bait treatments were significantly different within all four trapping sessions (*P* < 0.0001, Fig. 3, Table S1). In winter 2022, a total of 107 dung beetles were captured, while in winter 2023, 42 beetles were captured (Table S1). Notably, in winter 2022, dung baits attracted significantly more beetles than any of the chemical blends which were no more attractive than the unbaited control (*F*_*(*7,120)_ = 26.68, *P* < 0.0001), but in winter 2023 dung baits were not significantly more attractive than M2, M3, M5 blends (*F*_(7,114)_ = 5.18, *P* < 0.0001) (Fig. 3, Table S1). In spring (*F*_(7,152)_ = 11.04, *P* < 0.0001) and summer (*F*_(7,152)_ = 16.34, *P* < 0.0001) 2022, when beetles were most numerous, blends M1, M2 and M4 were as attractive as the dung bait and significantly more attractive than the unbaited control. Blend M2, which contained dimethyl sulphide and dimethyl disulphide in addition to six-compounds, overall was most consistently attractive mix, followed by M1 and M4, which had toluene in addition to the six-compounds. M6, the four-compound mix (dimethyl sulphide, dimethyl disulphide, toluene, and eucalyptol), was least attractive overall and never attracted significantly more beetles than did the unbaited control (Fig. 3, Table S1).

**Figure 4:**
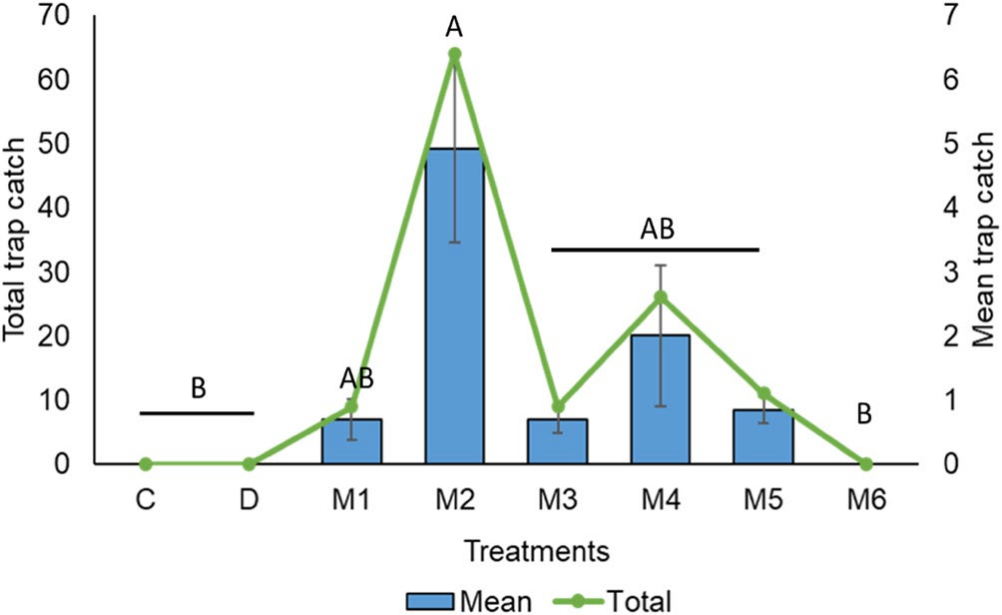
Mean trap catch (± SE) of *Omorgus* sp. caught in dung and synthetic baits. Different letters indicate significance among bait types at *P* < 0.001 as determined by Kruskal–Wallis one-way analysis of variance followed by Dunn’s means comparison test. *n* = 4. C—bait free, D—dung bait, M1—skatole + indole + phenol + butyric acid + butanone + *p*-cresol, M2—M1 + dimethyl sulphide + dimethyl disulphide, M3—M1 + eucalyptol, M4—M1 + toluene, M5—M1 + dimethyl sulphide + dimethyl disulphide + eucalyptol + toluene, M6—dimethyl sulphide + dimethyl disulphide + eucalyptol + toluene.

In addition to dung beetles, the carcass beetle *Omorgus* sp. (Coleoptera: Scarabaeoidea: Trogidae) was also collected in large numbers by the synthetic baits during the spring (2022) (Fig. 4). We were not able to identify these beetles beyond genus level. Among the synthetic baits, blend M2, which included DMS and DMDS in addition to six compounds (Table 1), attracted a significantly higher number of these beetles (*F*_(7,152)_ = 4.27, *P* = 0.0002) followed by M5, M4, M3 and M1. In contrast, dung baits, blend M6 and the control did not attract any *Omorgus* sp. at any time.

### Presence of field-tested VOCs in cattle dung bait headspace

**Figure 5:**
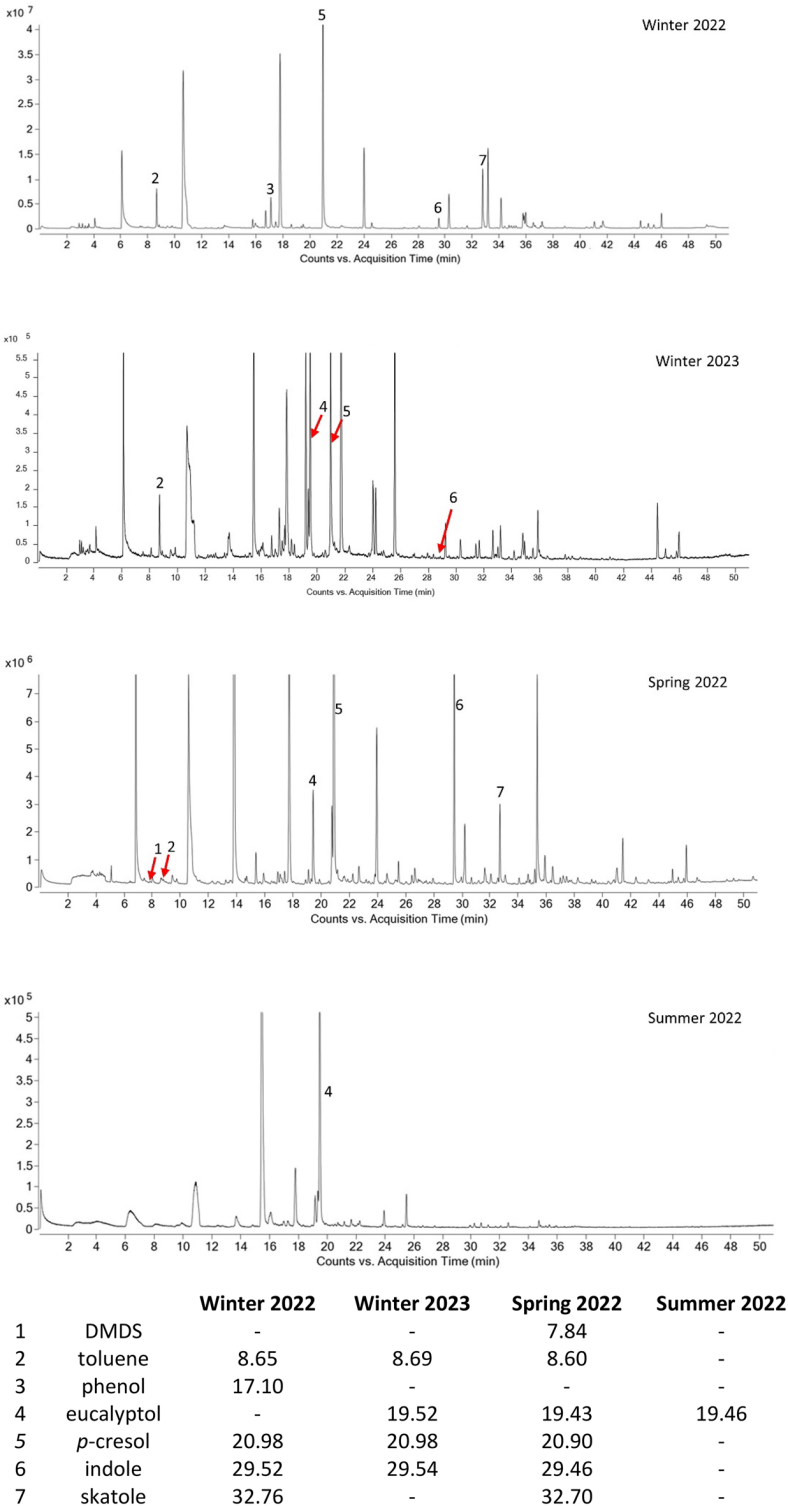
Qualitative representation of total ion chromatograms (TICs) and retention times (min ± 0.05) indicating differences in major VOCs detected in cattle dung headspace used as field baits. All annotated compounds were confirmed by standards injections.

The headspace volatilome of the cattle dung baits which was freshly collected just prior to each of the four trapping periods differed markedly (Fig. 5). Of the 10 compounds tested as lure constituents, seven were identified in the headspace of dung baits in at least one period (Figs. 5, S2). Dung used for 2022 winter field assays had relatively high levels of *p*-cresol, skatole, toluene and phenol. In contrast, the dung used in 2023 had toluene, eucalyptol, and *p*-cresol as most abundant compounds. Dung collected in spring (2022) had most compounds except phenol, with *p*-cresol, indole, skatole and eucalyptol predominating. The levels of DMDS and toluene were relatively low or zero. Notably, in dung used in summer (2022), eucalyptol was the only one of the 10 compounds tested that was detected via GC-MS.

## Discussion

The results of our field trapping experiments conducted over four seasonal episodes demonstrated that exotic dung beetles display differential attraction to the various baits. Dung baits consistently caught more dung beetles than did the unbaited control but the volatilome of the dungs available for use varied markedly among the four episodes, illustrating an inherent problem with the use of dung as a bait and the need for a synthetic bait that would standardise trapping across seasons. Previously, attraction of dung beetles to chemicals has rarely been studied^4,9^ and here, for the first time we present evidence for the field attraction of exotic dung beetles in Australia to volatile chemical blends. We used 10 compounds that previously were found to be electroantennographically active for dung beetles^10,43^. The present study also emphasizes the general attractiveness of dung beetles to volatile chemical blends that are known from the volatilome of dung.

The dung beetle species caught in greatest abundance overall was *O. taurus*, and was more abundantly caught in summer compared to the spring, which can be explained by spring emergence and breeding in this species that results in increasing beetle numbers in the summer^3^. This temporal trend was detected in the synthetic bait treatments as well as in dung treatment suggesting that these lures can capture such temporal effects. Trap catch in winter primarily consisted of *B. bison* and lower numbers of these species were caught in traps baited with synthetic lures compared to those of dung. Within a given trapping session, fluctuation in beetle numbers was more likely to be influenced by environmental factors such as temperature and wind conditions. Even though dung baits prepared from thawed frozen dung were considered attractive to dung-inhabiting fauna^49^, we used fresh dung in our study to simulate natural conditions. During preliminary volatile analysis, we observed changes in the chemical composition of the dung headspace between thawed frozen and fresh dung (data not shown). According to observations by Lumaret and Kirk, the attractiveness of a desiccated dung pat can possibly be partially restored by wetting the dung pat^3^. However, we replaced all the dung baits and concurrently all of the synthetic baits each day. The control treatment, consisting of a trap without any bait attracted only four beetles throughout the study, which validated the dung beetle trapping design.

Bait type significantly affected trap catch. The higher trap catch for dung baits can be attributed to the complex composition of dung volatilomes, which consist of numerous volatiles, whereas chemical blends employed in this study contained a restricted set of compounds, which were intended to mimic rather than replicate the chemical complexity of livestock dung volatilomes^4^. Our results highlight season-dependent variations in the attractiveness of synthetic baits suggesting that efficacy of using them as lures for dung beetles over time. Unlike previous studies, in our trapping program chemical baits were successful at attracting all of the same species that were attracted to dung baits^4,9^. Blend M1 was the base formulation for five of the six mixtures (M2–M5) (Table 1) and was comprised of six compounds which included characteristic dung volatiles used in previous studies^4,9^. Blend M2 was created by the addition of DMS and DMDS to M1. This change had seasonally variable impact and was observed to enhance the attractancy compared to M1 in winter and spring but had a negligible impact in summer (Fig. 3, Table S1). Blend M3 was made by the addition of eucalyptol to M1 and resulted in lower trap catches throughout all seasons, suggesting that the terpene has a nonspecific inhibitory effect on attractancy or somehow masked the presence of the more attractive compounds (Fig. 3, Table S1). Addition of toluene to M1 to give blend M4 increased the mean trap catch in spring and summer compared to M3, M5 and M6 (Fig. 3, Table S1). Blend M5 was a mixture of the 10 compounds used in the trial. In spring and summer, compared to M5, M1 was able to attract appreciably more dung beetles, which indicates that one or all added compounds (toluene, eucalyptol, DMS and DMDS) in M5 may have reduced the attractancy for beetles. Blend M6, differed from all other blends as it did not use M1 as a base. It consisted of four compounds, toluene, eucalyptol, DMS and DMDS, and displayed the lowest attractancy of all the blend used in the study.

Volatilome analysis of field baits revealed that the relative abundance of 10 compounds in the dung headspace may have influenced the attraction of dung beetles to dung and synthetic baits. In winter 2022, dung baits showed significant attraction to the dominant species (*B. bison*)*,* potentially due to the release of *p*-cresol, indole, skatole, phenol, and toluene in quantities favourable to beetles. Similarly, dung baits used in winter 2023 contained toluene, *p*-cresol, and abundant eucalyptol (Fig. 5). In spring, the cattle dung volatilome contained six of the 10 compounds used in the study, including *p*-cresol, indole and skatole, which we propose accounts for the attractiveness of the dung baits. Eucalyptol dominated the dung bait composition in summer, and blends M1, M2 and M4, none of which contained eucalyptol, attracted relatively greater number of beetles. It seems probable that the presence of high levels of eucalyptol in dung during spring, summer and winter (2023) might act as a repellent for dung beetles by decreasing the attractiveness of dung baits. Eucalyptol has been reported to be toxic to insects and has been used in insecticides^50–53^. Nonetheless, *p*-cresol, indole and skatole were detected in dung, and are known to be attractive to dung beetles^4,10,19^. We did not detect butanone and butyric acid in the headspace of any dung bait used despite their importance as odour cues for dung beetles^9,16^. Overall, these observations support the role of skatole, indole, phenol, and *p*-cresol in M1 as attractant for dung beetles. In fact, these compounds have been detected in male abdominal secretion in the dung beetle *Kheper* spp.^54^. Similarly, DMS, DMDS in blend M2 and toluene in blend M4 may have enhanced the attractiveness for dung beetles.

We chose to evaluate chemical mixtures in this experiment rather than individual compounds based on previous studies, as the effects of mixtures can be different to the sum of the individual parts^4,9,10^. Because of the numerous volatiles present in the dung headspace and their variable concentrations^4,5,10,11^, a huge number of possible combinations of compounds exists. Therefore, we limited our study to 10 EAG-active compounds we had identified in our previous study^10,43^ and six blends were tested. Our results have provided information illuminating several aspects of dung beetle chemical ecology. During preliminary studies, we observed crystallisation of compounds due to low winter temperatures, reducing the amount of these molecules in the vapour phase and presumably impacting attraction of dung beetles to chemical lures. There was no evidence that any of the species exclusively preferred one synthetic bait over the other, but the abundance of a given species caught in a trap could vary significantly between synthetic baits. Furthermore, it has been suggested that early colonising dung beetles can alter the dung headspace composition by releasing pheromones^12^. For instance, early colonising males of the dung beetle *Aphodius fossor* have been found to attract females, while early colonising females tend to repel late colonising females thereby reducing competition from conspecifics^13^. Pheromone-induced interplay between dung beetles has not been studied except on very few occasions^12,13,55^.

Although scarabaeid dung beetles were the primary focus of our study, *Omorgus* sp. was also attracted to our synthetic baits showing their preference for chemical blends. Beetles of the family Trogidae are important as decomposers in many ecosystems and are known to be generalist feeders^56,57^. Specially in the field of forensic entomology, *Omorgus* spp. have a high value as an indicator species as they feed on keratin found in hair and skin^58–60^. They have occasionally been found in association with dung beetles colonising dung and carrion^3,61–64^. *Omorgus suberosus* was found in pitfall traps baited with cow dung, pig manure, human faeces and carrion^57^. *Omorgus suberosus*, is a facultative predator on the eggs of orthopterans and reptiles in Mexico, especially ‘vulnerable’ olive ridley turtles. The antenna of this insect was found to respond significantly to indole, DMS and DMDS, with indole found to be more attractive in the field study^56,65^. The attraction of *Omorgus* sp. during our trapping, especially towards blend M2, which included DMS and DMDS alongside indole, supports the purported behavioural activity of these volatiles in attracting trogid beetles. Interestingly, there was an apparent increase in the attractiveness of synthetic baits containing DMDS and DMS, compared to the M1 blend that contained only the six-compound mix including indole. The attractiveness of trogid beetles to DMS and DMDS may be explained by the fact that those two compounds are being released from vertebrate cadavers/carrion^66,67^. The presence of eucalyptol or toluene appeared to have a negative impact on the attractancy of DMS and DMDS to *Omorgus* sp, indicated by lower trap catch for blends M3, M4 and M5 compared to blend M2 in which these compounds were not present. These data may be of use for developing a trapping method to control predatory *Omorgus* sp.

When using several compounds in a blend, it is important to note that odour identity and odour intensity may impact the perception of odours by insects. Based on the detection threshold of the insects, it has been shown that odour intensity can affect its ability to differentiate among odorants, despite the molecular properties of the odorant molecules^68^. In some cases, for the same odorant, olfactory receptors can exhibit either an elevated or depressed affinity depending on whether the molecule has a lower or a higher concentration^69^. In our experiments, we used pure compounds at equal amounts based on previous studies^4,9^ as the ability of dung beetles to discriminate odour molecules at different concentrations or the optimum perception conditions of odours has not yet been determined. In nature, odorants can occur in odour bouquets at different ratios contributing to scent recognition and discrimination^70^. However, the ability of insects to discriminate among odorants tends to increase at higher odorant concentrations^71^. In addition, background odour can also interfere with the sensitivity of the insect olfactory system^72,73^ and extended field exposure of mixtures may cause a loss of attractiveness due to evaporation of volatile compounds. Therefore, research is required to evaluate a range of concentrations and ratios of test compounds, field volatilisation rates at different ambient temperatures, masking effects, involvement of background odour, and dung beetle detection thresholds for odorants to understand how these factors affect attraction to chemical baits in the field.

To keep trapped insects alive, which is a primary objective when trapping insects for rearing or redistribution, it is important not to use toxic liquids as the trapping medium. From our observations, the current trap design using water as the trapping medium seem ideal for preventing the escape of trapped dung beetles without killing them. Another advantage of using water as the trapping medium is that it prevents the release of odours from putrefying insects that would likely interfere with the bait attractiveness^74^. A standardised monitoring method is also fundamental when assessing the seasonal and geographical abundance of dung beetles as the dung volatilomes vary across seasons as revealed by dung analysis (Figs. 5, S2). Our results pinpoint the importance of using behaviourally active compounds in synthetic baits as the basis of a consistent and more reliable sampling procedure. Currently there is no evidence that dung beetle olfactory preference varies geographically, especially in the case of introduced species occurring outside their native distribution. Thus, chemical baits offer the potential to permit trapping of dung beetles in a standardised, consistent fashion with materials that are easier to handle, are cleaner and do not pose a biosecurity risk as does livestock dung.

Our results suggest that using chemical baits for trapping dung beetles shows considerable promise. Previously, we provided evidence for the presence of olfactory receptors for the tested compounds on dung beetle antennae^43^. Here, we establish evidence for the potential role of these compounds, including skatole, indole, phenol, butyric acid, butanone, and *p*-cresol, as attractants for trapping dung beetles in the field. Additionally, we have shown that other compounds (e.g., DMS, DMDS, toluene and eucalyptol) modify the attraction of dung beetles to the primary attractants. It is important to note that our aim was not to formulate a lure that replicates dung odour, which would be difficult depending on the unique chemistry of dung, but to take a step towards understanding the chemical ecology of dung attraction and establishing the efficacy of using chemical baits for trapping dung beetles. This was achieved by testing previously reported attractant chemicals in various combinations with other compounds we have found in attractive dung and testing them across time against a range of dung beetles. Additional studies involving other dung beetle species in other locations and additional formulations of lures are required to evaluate the generalisability of our findings.

### Supplementary Information

Supplementary Information.

## Data Availability

All data generated or analysed during this study are included in this published article.
