# Supplementary material for: Field evaluation of electrophysiologically-active dung volatiles as chemical lures for trapping of dung beetles
Source: Sci Rep. 2024 Jan 5;14:584. doi: 10.1038/s41598-023-50079-3 (PMC10770360; doi:10.1038/s41598-023-50079-3)
Supplement: Supplementary file 1 — Supplementary Information. [file 41598_2023_50079_MOESM1_ESM.docx]

Field evaluation of electrophysiologically-active dung volatiles as chemical lures for trapping of dung beetles

# Nisansala N. Perera^1,2^, Russell A. Barrow ^1^, Paul A. Weston ^1^, Leslie A. Weston^1,2^, and Geoff M. Gurr ^1,3, *^

# Supplementary Materials

**Fig. S1** Line graphs showing the frequency abundance of the beetles attracted to dung and synthetic baits during winter (2022 and 2023), spring, and summer seasons. **C**- bait free, **D**- dung bait, **M1**- skatole + indole + phenol + butyric acid + butanone + p-cresol, **M2**- M1 + dimethyl sulphide + dimethyl disulphide, **M3**- M1 + eucalyptol, **M4**- M1 + toluene, **M5**- M1 + dimethyl sulphide + dimethyl disulphide + eucalyptol + toluene, **M6**-dimethyl sulphide + dimethyl disulphide + eucalyptol + toluene.


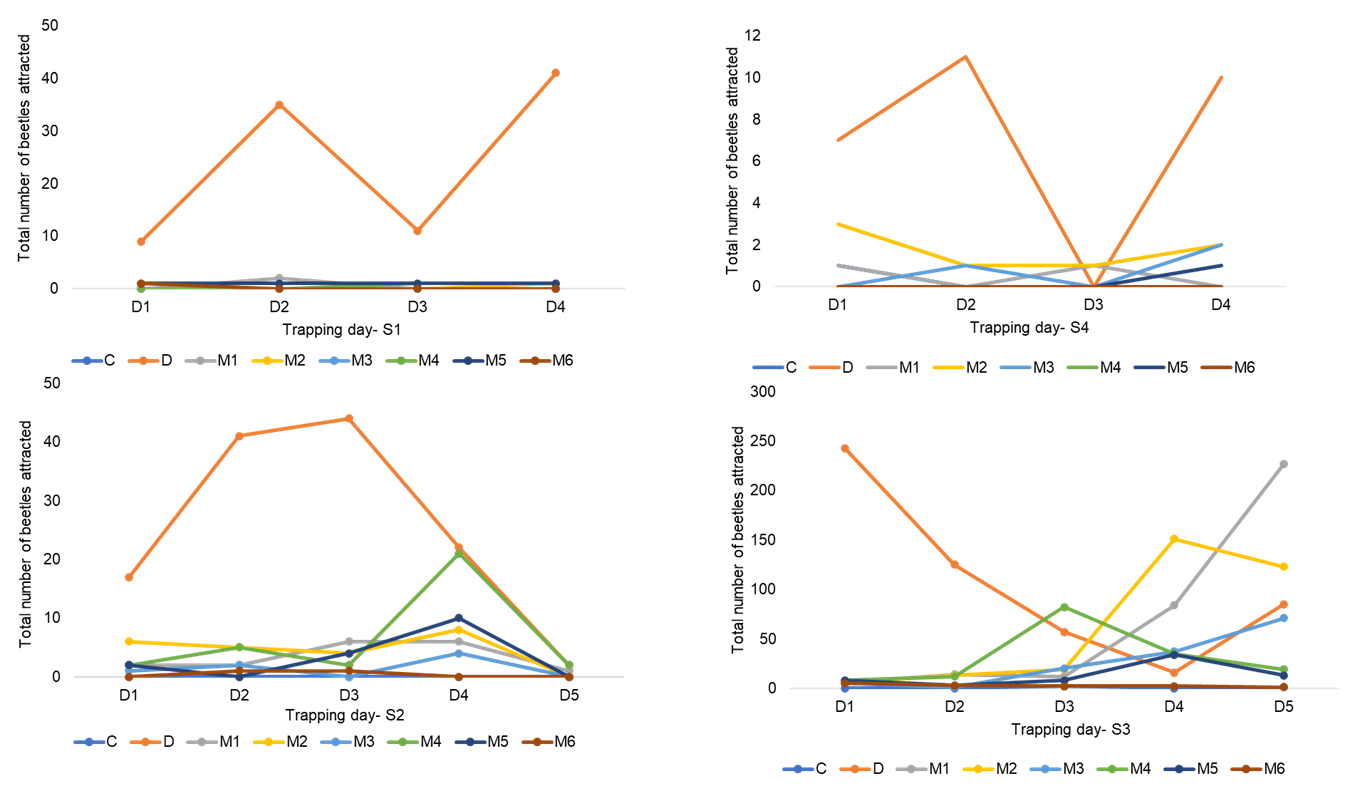


**Winter 2022**

**Spring**

**Summer**

**Winter 2023**

|  |
| --- |
|  |

**Table S1** Total and mean trap catch for each bait type across four seasons. The number of replicates for each bait type deployed was 16 in winter and 20 in spring and summer. For all four sessions P < 0.001 as determined by Kruskal-Wallis one-way nonparametric analysis of variance. Means in a column followed by the same superscripted upper-case letters are not significantly different as determined by Dunn's all pairwise comparison test. **C**- bait free, **D**- dung bait, **M1**- skatole + indole + phenol + butyric acid + butanone + p-cresol, **M2**- M1 + dimethyl sulphide + dimethyl disulphide, **M3**- M1 + eucalyptol, **M4**- M1 + toluene, **M5**- M1 + dimethyl sulphide + dimethyl disulphide + eucalyptol + toluene, **M6**-dimethyl sulphide + dimethyl disulphide + eucalyptol + toluene.

|  | **Winter 2022** | | **Winter 2023** | | **Spring 2022** | | **Summer 2022** | |
| --- | --- | --- | --- | --- | --- | --- | --- | --- |
|  | **Total** | **Mean ± SE** | **Total** | **Mean ± SE** | **Total** | **Mean ± SE** | **Total** | **Mean ± SE** |
| **Control** | 0 | 0.000 ± 0.00^B^ | 1 | 0.063 **±** 0.06^B^ | 0 | 0.00 ± 0.0^D^ | 3 | 0.15 ± 0.1^D^ |
| **Dung** | 96 | 6.000 ± 1.20^A^ | 28 | 1.75 ± 0.55^A^ | 126 | 6.30 ± 1.5^A^ | 527 | 26.30 ± 5.9^A^ |
| **M1** | 2 | 0.063 ± 0.06^B^ | 2 | 0.125 ± 0.09^B^ | 17 | 0.85 ± 0.1^ABC^ | 343 | 17.15 ± 6.8^AB^ |
| **M2** | 2 | 0.125 ± 0.08^B^ | 7 | 0.500 ± 0.16^AB^ | 23 | 1.25 ± 0.2^AB^ | 312 | 15.55 ± 4.1^AB^ |
| **M3** | 0 | 0.000 ± 0.00^B^ | 3 | 0.200 ± 0.14^AB^ | 7 | 0.35 ± 0.1^BCD^ | 134 | 6.70 ± 2.1^BC^ |
| **M4** | 2 | 0.125 ± 0.08^B^ | 0 | 0.000 ± 0.00^B^ | 32 | 1.55 ± 0.5^ABCD^ | 156 | 7.90 ± 2.8^AB^ |
| **M5** | 4 | 0.310 ± 0.15^B^ | 1 | 0.133 ± 0.09^AB^ | 16 | 0.90 ± 0.2^BCD^ | 66 | 3.30 ± 1.5^BCD^ |
| **M6** | 1 | 0.063 ± 0.06^B^ | 0 | 0.000 ± 0.00^B^ | 2 | 0.10 ± 0.1^CD^ | 13 | 0.65 ± 0.1^CD^ |

| 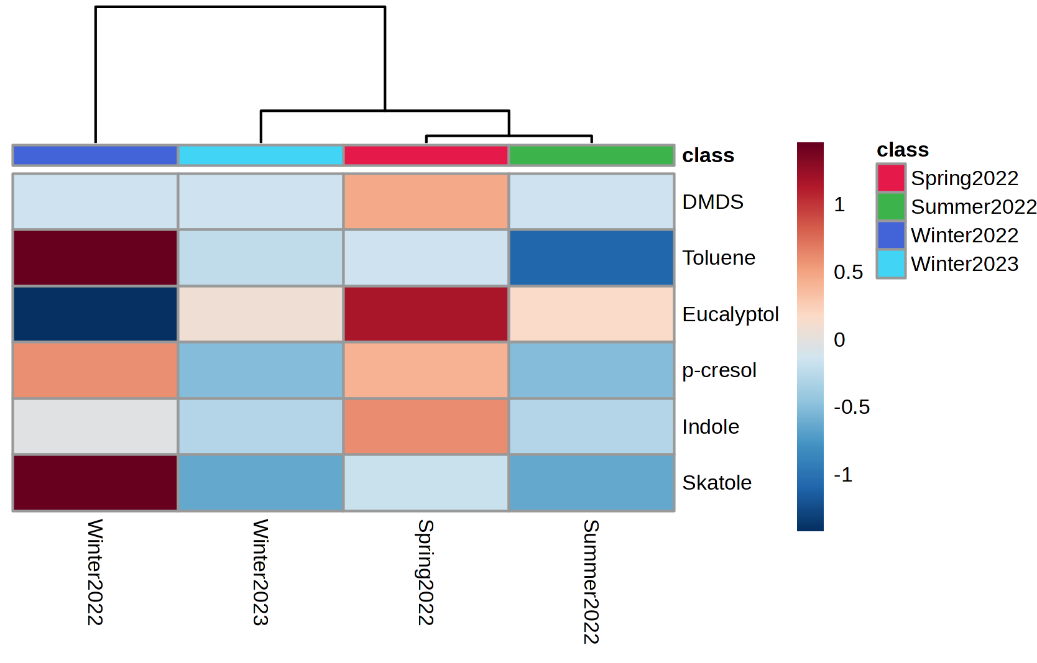  **Fig. S2** Hierarchical clustering heat map showing the presence and relative abundance of major dung volatiles tested as synthetic baits, in cattle dung baits headspace. |
| --- |

**Table S2** Chemicals, CAS numbers and purity used in the study.

| **Chemical** | **CAS number** | **Purity %** |
| --- | --- | --- |
| phenol | 108-95-2 | 99.0-100.5 |
| skatole | 83-34-1 | 98 |
| indole | 120-72-9 | ≥ 99 |
| butanone | 78-93-3 | ≥ 99.0 |
| butyric acid | 107-92-6 | ≥ 99 |
| *p*-cresol | 106-44-5 | 99 |
| eucalyptol | 470-82-6 | 99 |
| toluene | 108-88-3 | 99.9 |
| dimethyl sulphide | 75-18-3 | ≥ 99.0 |
| dimethyl disulphide | 624-92-0 | ≥ 99.0 |

**Table S3:** Species composition and abundance attracted to dung and synthetic baits in different trapping sessions. Here S1- winter 2022, S2- Spring, S3- Summer, S4- Winter 2024. **C**- bait free, **D**- dung bait, **M1**- skatole + indole + phenol + butyric acid + butanone + p-cresol, **M2**- M1 + dimethyl sulphide + dimethyl disulphide, **M3**- M1 + eucalyptol, **M4**- M1 + toluene, **M5**- M1 + dimethyl sulphide + dimethyl disulphide + eucalyptol + toluene, **M6**-dimethyl sulphide + dimethyl disulphide + eucalyptol + toluene.

|  | *B. bison* | | | *O. taurus* | | *E. pallipes* | | *E. fulvus* | | *E. africanus* | *A. fimetarius* | *O. alexis* | *O. pentacanthus* | | *D. gazella* | | *O.dandalu* | *Omorgus* sp. | |
| --- | --- | --- | --- | --- | --- | --- | --- | --- | --- | --- | --- | --- | --- | --- | --- | --- | --- | --- | --- |
|  | S1 | S2 | S4 | S2 | S3 | S2 | S3 | S2 | S3 | S3 | S2 | S3 | S2 | S4 | S2 | S3 | S3 | S2 | S3 |
| ***C*** | 0 | 0 | 1 | 0 | 2 | 0 | 0 | 0 | 0 | 0 | 0 | 1 | 0 | 0 | 0 | 0 | 0 | 0 | 0 |
| ***D*** | 96 | 0 | 27 | 115 | 407 | 6 | 1 | 1 | 44 | 1 | 4 | 0 | 0 | 1 | 0 | 2 | 72 | 0 | 0 |
| ***M1*** | 2 | 0 | 2 | 11 | 337 | 1 | 0 | 0 | 4 | 0 | 1 | 1 | 0 | 0 | 4 | 1 | 0 | 9 | 0 |
| ***M2*** | 2 | 0 | 7 | 15 | 306 | 0 | 0 | 0 | 6 | 0 | 1 | 0 | 0 | 0 | 6 | 0 | 0 | 59 | 5 |
| ***M3*** | 0 | 0 | 3 | 4 | 133 | 0 | 0 | 0 | 1 | 0 | 1 | 0 | 0 | 0 | 2 | 0 | 0 | 8 | 1 |
| ***M4*** | 2 | 1 | 0 | 22 | 152 | 3 | 1 | 1 | 3 | 0 | 1 | 0 | 1 | 0 | 4 | 0 | 0 | 25 | 1 |
| ***M5*** | 4 | 0 | 1 | 13 | 62 | 0 | 1 | 1 | 1 | 0 | 0 | 0 | 0 | 0 | 2 | 2 | 0 | 8 | 3 |
| ***M6*** | 1 | 0 | 0 | 1 | 10 | 0 | 2 | 0 | 0 | 0 | 0 | 0 | 0 | 0 | 1 | 0 | 1 | 0 | 0 |
